# Supplementary material for: The interactome of intact mitochondria by cross-linking mass spectrometry provides evidence for coexisting respiratory supercomplexes
Source: Mol Cell Proteomics. 2017 Dec 8;17(2):216–32. doi: 10.1074/mcp.RA117.000470 (PMC5795388; doi:10.1074/mcp.RA117.000470)
Supplement: Supplemental Data [file supp_RA117.000470_133922_0_supp_23978_fzffwf.zip › spectra_annotation/mito_DR_spectra_annotation/211-1-12-1-5-1.pdf]

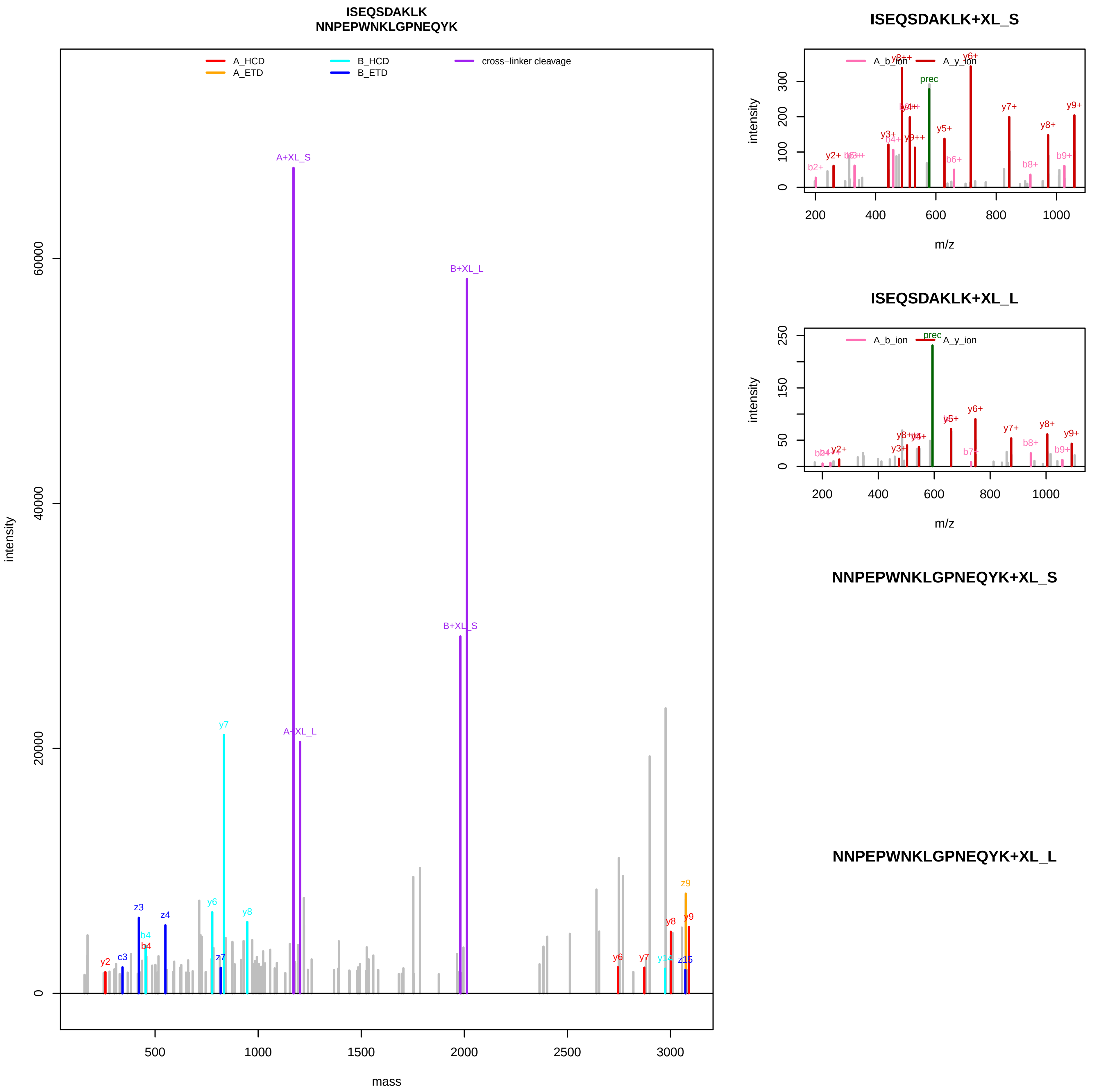
ISEQSDAKLK
NNPEPWNKLGPNEQYK
A_HCD
A_ETD
B_HCD
B_ETD
cross−linker cleavage
A+XL_S
B+XL_L
B+XL_S
A+XL_L
y7
y6
y8
z3
z4
c3
b4
y2
z7
z9
y9
y8
y6
y7
z15
intensity
mass
ISEQSDAKLK+XL_S
A_b_ion
A_y_ion
prec
y7+
y8+
y9+
y5+
y3+
y2+
b2+
b6+
b8+
b9+
m/z
ISEQSDAKLK+XL_L
NNPEPWNKLGPNEQYK+XL_S
NNPEPWNKLGPNEQYK+XL_L
